# Compound‐Heterozygous *PLVAP* Missense Variants Associated With Attenuated Protein‐Losing Enteropathy (DIAR10)

**DOI:** 10.1111/cge.70258

**Published:** 2026-10-07

**Authors:** Jana Zídková, Dagmar Procházková, Petra Konečná, Kamila Réblová, Tereza Kramářová, Johana Kopčilová, Magdaléna Soukup Vodičková, Romana Borská, Lenka Fajkusová

**Affiliations:** ^1^ Centre of Molecular Biology and Genetics University Hospital Brno and Masaryk University Brno Czech Republic; ^2^ Department of Pediatrics University Hospital Brno and Masaryk University Brno Czech Republic; ^3^ CEITEC ‐ Central European Institute of Technology, Masaryk University Brno Czech Republic; ^4^ National Centre for Biomolecular Research, Faculty of Science Masaryk University Brno Czech Republic

**Keywords:** DIAR10, endothelial diaphragm, PLVAP, protein‐losing enteropathy

## Abstract

A three‐year‐old girl with attenuated protein‐losing enteropathy carried compound‐heterozygous missense *PLVAP* variants inherited from unaffected non‐consanguineous parents. The case supports residual PLVAP function in missense‐associated DIAR10 and expands its genetic and clinical spectrum.

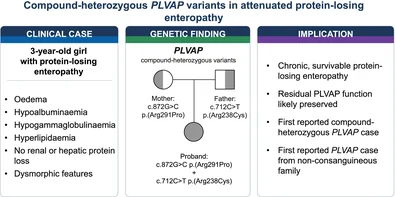

Protein‐losing enteropathy (PLE) results from excessive gastrointestinal loss of plasma proteins. Biallelic pathogenic *PLVAP* variants cause diarrhea 10, protein‐losing enteropathy (DIAR10), an ultra‐rare disorder of endothelial fenestral and intestinal lacteal diaphragms. Published molecularly confirmed cases have involved homozygous variants, usually in consanguineous families. Truncating variants cause severe neonatal or infantile disease and early death [1, 2, 3], whereas homozygous p.(Leu34Pro) was associated with an attenuated phenotype [4]. We report a Czech child with DIAR10 caused by compound heterozygous *PLVAP* missense variants.

The patient was a 3‐year‐old girl born to non‐consanguineous parents with no relevant family history. Pregnancy was complicated by intrauterine growth restriction and anhydramnios. Delivery was performed at 37 weeks for impending fetal hypoxia; birth weight and length were 1850 g and 43 cm. The neonatal course included hypotonia, hypoxia, respiratory distress requiring intubation, delayed meconium passage, abdominal distension, parenteral nutrition, polycythaemia and thrombocytopenia requiring transfusion support.

At 15 months, she presented with oedema of the legs, abdomen and eyelids. Laboratory tests showed severe hypoalbuminaemia (nadir 15 g/L), hypogammaglobulinaemia (IgG 1.32 g/L; IgA 0.53 g/L; normal IgM), hypocalcaemia, vitamin D deficiency and marked dyslipidaemia (cholesterol 11.1 mmol/L; triglycerides 10.0 mmol/L). Liver enzymes, bilirubin and acid–base status were normal; 24‐h urinary protein excretion was not increased (0.1 g/24 h), arguing against hepatic or renal protein loss. Abdominal ultrasound showed generalised fluid infiltration. Although intestinal biopsy was not performed, the biochemical pattern favoured an endothelial or intestinal protein‐leak mechanism. Notably, she had no diarrhoea at presentation and has not developed diarrhoea during follow‐up.

At 32 months, length and weight were below the 3rd percentile. Dysmorphic features included triangular face, arched forehead, broad nasal bridge, deep‐set eyes, low‐set ears, narrow lips, elongated philtrum, small chin and crescent‐shaped toes (Figure 1). Neurological examination showed triparetic cerebral palsy, moderate intellectual disability and expressive language delay, considered more likely secondary to perinatal hypoxic‐ischaemic complications than to primary PLVAP dysfunction. At last follow‐up, aged 3 years, she was alive with a chronic, clinically stable course under supportive management.

**FIGURE 1:**
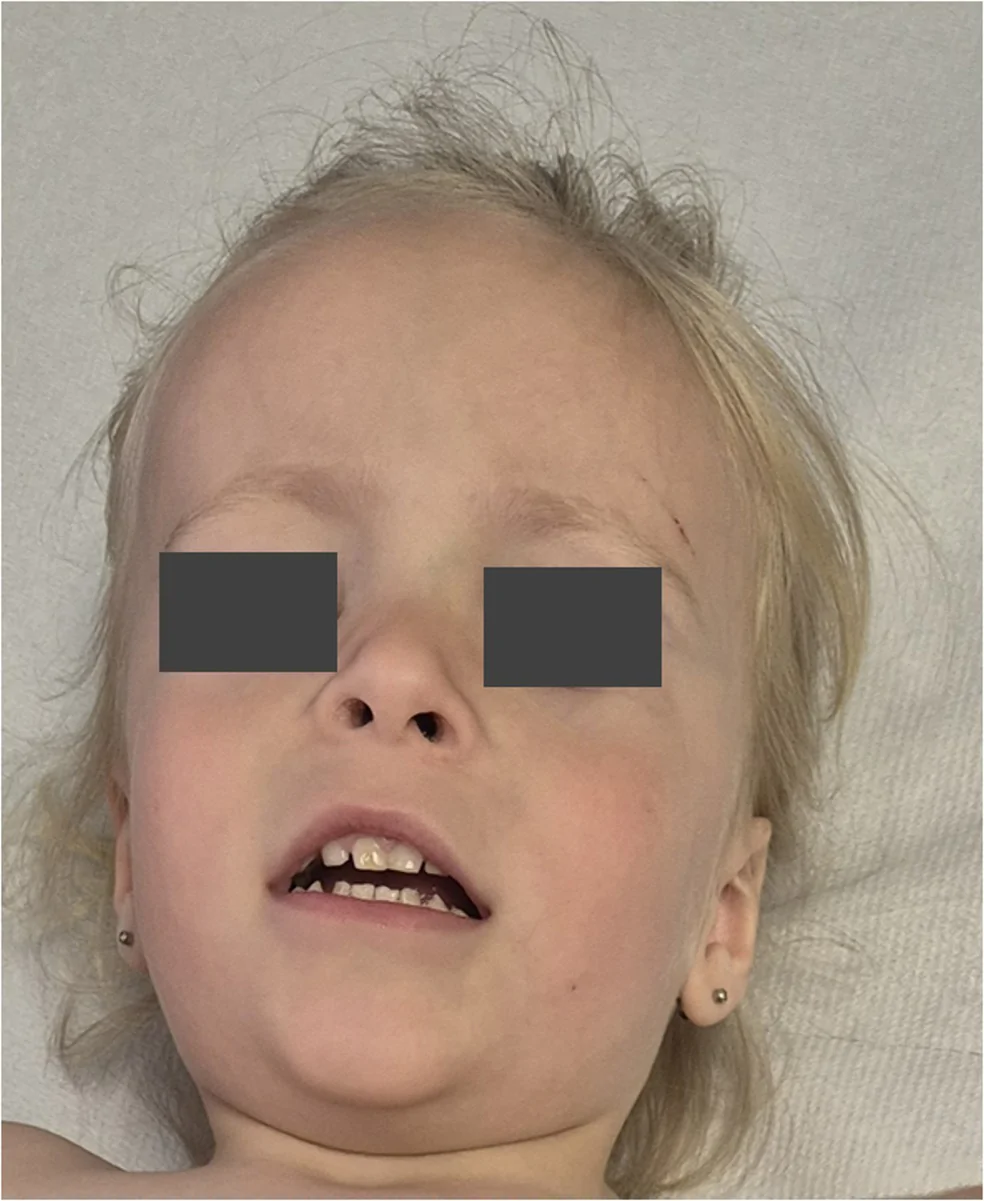
Clinical appearance showing a triangular face, arched forehead, broad nasal bridge, low‐set ears, narrow lips, elongated philtrum and small chin. [Colour figure can be viewed at wileyonlinelibrary.com]

Trio whole‐exome sequencing aligned to GRCh38 identified two rare *PLVAP* variants (NM_031310.3) in compound heterozygosity: c.712C>T p.(Arg238Cys) and c.872G>C p.(Arg291Pro). Segregation confirmed inheritance in *trans*. Both variants are currently classified as variants of uncertain significance according to ACMG/AMP criteria. The c.712C>T variant has a minor allele frequency (MAF) of 0.0000093 and a CADD score of 22.8, whereas the c.872G>C variant has a MAF of 0.00000085 and a CADD score of 24.2. The combination of the biallelic *PLVAP* genotype with profound hypoalbuminaemia and hypogammaglobulinaemia, marked hyperlipidaemia, normal hepatic enzymes and absent renal protein loss were highly suggestive of *PLVAP*‐associated PLE. Both substitutions are located within the coiled‐coil region of PLVAP [5] and affect the extracellular region required for PLVAP oligomerisation and diaphragm formation and are predicted to reduce, rather than abolish, PLVAP function.

The published DIAR10 spectrum is limited. Reported disease‐associated *PLVAP* variants include p.(Gln330*) [1], p.(Arg358*) [2], p.(Ala114Cysfs*9) [3] and p.(Leu34Pro) [4]. Patients with homozygous null variants typically developed neonatal or early infantile oedema, diarrhoea, profound hypoalbuminaemia, hypogammaglobulinaemia, dyslipidaemia, electrolyte disturbance, recurrent infections or thrombotic complications, with high mortality. In contrast, our patient's survivable course resembles the attenuated spectrum reported for biallelic missense variation and supports residual PLVAP activity.

This case expands the recognised DIAR10 spectrum. To our knowledge, it is the first published *PLVAP*‐associated PLE case from a non‐consanguineous family, the first compound‐heterozygous case and a further example of chronic, survivable disease associated with missense alleles. It also illustrates variant interpretation in ultra‐rare disorders: without functional data, segregation, a specific biochemical signature and a coherent structural mechanism can provide diagnostic support, although the formal VUS classification should be retained. *PLVAP* should be considered in infants and toddlers with unexplained oedema, hypoalbuminaemia, hypogammaglobulinaemia and hyperlipidaemia after exclusion of renal and hepatic causes.

## Funding

This work was supported by the Ministry of Health, Czech Republic (FNBr, 65269705). We acknowledge the Core Facility Genomics, NCLG research infrastructure (LM2018132, MEYS CR) and e‐INFRA CZ (ID: 90254).

## Ethics Statement

Approved by the Ethical Committee of the University Hospital Brno (ID 4‐326/23/5). Written informed consent to participate and for publication of the patient's image was obtained from the parents.

## Conflicts of Interest

The authors declare no conflicts of interest.

## Data Availability

Data are available at the Centre of Molecular Biology and Genetics, University Hospital Brno, Brno, Czech Republic.

## References

[1] I. J. Broekaert , K. Becker , I. Gottschalk , et al., “Mutations in Plasmalemma Vesicle‐Associated Protein Cause Severe Syndromic Protein‐Losing Enteropathy,” Journal of Medical Genetics 55 (2018): 637–640. 10.1136/jmedgenet-2018-105262 29661969

[2] A. Elkadri , C. Thoeni , S. J. Deharvengt , et al., “Mutations in Plasmalemma Vesicle Associated Protein Result in Sieving Protein‐Losing Enteropathy Characterized by Hypoproteinemia, Hypoalbuminemia, and Hypertriglyceridemia,” Cellular and Molecular Gastroenterology and Hepatology 1 (2015): 381–394.e7. 10.1016/j.jcmgh.2015.05.001 PMC4507283 26207260

[3] O. Gorukmez , O. Gorukmez , and K. Demiroren , “NovelPLVAPMutation in Protein Losing Enteropathy,” Fetal and Pediatric Pathology 38 (2019): 534–537. 10.1080/15513815.2019.1627624 31215290

[4] A. Kurolap , O. Eshach‐Adiv , C. Gonzaga‐Jauregui , et al., “Establishing the Role ofPLVAPin Protein‐Losing Enteropathy: A Homozygous Missense Variant Leads to an Attenuated Phenotype,” Journal of Medical Genetics 55 (2018): 779–784. 10.1136/jmedgenet-2018-105299 29875123

[5] T. H. Chang , F. L. Hsieh , X. Gu , et al., “Structural Insights Into Plasmalemma Vesicle‐Associated Protein (PLVAP): Implications for Vascular Endothelial Diaphragms and Fenestrae,” Proceedings of the National Academy of Sciences of the United States of America 120, no. 14 (2023): e2221103120. 10.1073/pnas.2221103120 PMC10083539 36996108

